# Efficacy and Tolerance of Synthetic Cannabidiol for Treatment of Drug Resistant Epilepsy

**DOI:** 10.3389/fneur.2019.01313

**Published:** 2019-12-10

**Authors:** Kerstin A. Klotz, Daniel Grob, Martin Hirsch, Birgitta Metternich, Andreas Schulze-Bonhage, Julia Jacobs

**Affiliations:** ^1^Faculty of Medicine, Freiburg Epilepsy Center, Medical Center - University of Freiburg, Freiburg im Breisgau, Germany; ^2^Berta-Ottenstein-Programme, Faculty of Medicine, University of Freiburg, Freiburg im Breisgau, Germany; ^3^Department of Neuropediatrics and Muscle Disorders, Faculty of Medicine, Center for Pediatrics, Medical Center - University of Freiburg, Freiburg im Breisgau, Germany

**Keywords:** epilepsy, cannabidiol, open label, pharmacotherapy, adverse events, cannabinoids, antiepileptic drug

## Abstract

**Objective:** Controlled and open label trials have demonstrated efficacy of cannabidiol for certain epileptic encephalopathies. However, plant derived cannabidiol products have been used almost exclusively. Efficacy of synthetically derived cannabidiol has not been studied before. The objective of this study was to evaluate tolerability and efficacy of synthetic cannabidiol in patients with pharmacoresistant epilepsy.

**Methods:** In this prospective, open-label study (DRKS00013177), patients with pharmacoresistant epilepsy received synthetic cannabidiol in addition to their previously stable anticonvulsive treatment. Starting dose was 5 mg/kg/day, up-titrated to a maximum of 50 mg/kg/day. Primary efficacy endpoint was monthly frequency of motor seizures at 3 months.

**Results:** Between April 2017 and May 2019, 35 patients were enrolled in the study. Mean age was 19.7 years (SD 14.6). Median motor seizure frequency decreased from 21.8 (IQR 7.5–52.5) seizures per month at baseline to 8.5 (IQR 3.7–28.3, *p* < 0.001) at 3 months, effect not influenced by AED changes and drop-outs. Adjusted percentage reduction was 40.0% (IQR 18.2–58.5). Adverse events (AE) were reported in 25 patients (71.4%), most frequently somnolence (40%), diarrhea (34.3), and loss of appetite (20%). Two patients (5.7%) discontinued treatment due to AE. Median (range) of treatment duration was 321 days (range 36–824). With ongoing treatment up to date in 21 patients (60%).

**Conclusion:** Efficacy and tolerance in our study of synthetic CBD treatment in pharmacoresistant epilepsy is similar to open label studies using plant derived CBD. Regarding economic and ecological aspects, synthetic cannabidiol might be a reasonable alternative to plant derived cannabidiol.

## Introduction

Over the last decade, the therapeutic use of cannabidiol (CBD) in intractable epilepsies has increased considerably (1). Its anticonvulsant properties have been shown in several animal models for acute and chronic epilepsy (2). Recent randomized, controlled trials have demonstrated that CBD is superior to placebo in seizure reduction in children with Dravet syndrome and patients with Lennox-Gastaut syndrome (3–5). In addition, open label studies indicate that cannabidiol has anticonvulsive properties in a broader range of epilepsy syndromes and etiologies (6). In most studies, 10% solutions of purified CBD are used, in some with additional small amounts of delta-9-tetrahydrocannabinol (THC) (7). Regardless of compositions, all studied preparations contain plant derived CBD (8). Recently, the first pharmaceutical formulation of highly purified, plant derived CBD has been approved by the US Food and Drug Administration (9). Single molecule cannabinoid drug development is a different approach where pharmaceutical-grade synthetically derived substances are used (10). Easier quality control, unlimited production possibilities and reduced environmental impact are advantages of synthetically derived cannabinoids and support further investigations of its therapeutic use. Synthetic CBD is a (+)-enantiomer of the (–)-natural CBD. Since the chemical structure is otherwise identical, similar efficacy and tolerance are to be expected (11). However, besides one phase II study and one study using transdermal application, to our knowledge no studies using synthetic CBD in pharmacoresistant epilepsies have been published (12, 13).

The objective of this study was to evaluate the long-term safety, tolerability, and efficacy of synthetic cannabidiol in children and adults with pharmacoresistant epilepsy.

## Materials and Methods

### Patients

Patients with pharmacoresistant epilepsy as defined by the International League Against Epilepsy, medicated with at least one anticonvulsive drug (AED) at a stable dose for 4 weeks pre-intervention, stable ketogenic diet/vagal nerve stimulation device settings for at least 4 weeks pre-intervention and willingness of patients/caregivers to comply with seizure diary were eligible for inclusion. Exclusion criteria were current treatment with cannabis-based products, pregnancy or unstable hepatic, or renal disease.

### Standard Protocol Approvals, Registrations, and Patients Consent

The trial was approved by the institutional research ethics board (397/17) and registered (DRKS00013177). All patients or parents/legal representatives provided written informed consent and assent according to patients' physical and mental capability before trial onset.

### Trial Design

This prospective, open-label, observational study was conducted at the University Epilepsy Center in Freiburg, Germany since November 2017; data cut was August 2019. Patients were prospectively followed by a pediatric neurologist or by a neurologist. Visits were scheduled at baseline and at 3, 6, and 12 months of treatment. Patients received a pharmaceutical formulation of synthetic CBD, manufactured by THC Pharm GmbH/Germany, in a 100 mg per mL MCT-oil-based oral solution, according to national drug-preparation regulations. An internal quality control at final solution level was performed at our center. CBD was administered orally in addition to the baseline antiepileptic drug regimen at a starting dose of 5 mg/kg/days divided into two daily doses. Patients were advised to take CBD with fatty meals. Dosage was up-titrated by 2–5 mg/kg/days up to 18–20 mg/kg/days over 14–21 days. If no effect was observed, dosage could be increased further up to 50 mg/kg/days. Concomitant AEDs were reviewed at each clinic visit. For the first 3 months of cannabidiol treatment, efforts were made to keep concomitant doses of antiepileptic drug constant. However, if addition of cannabidiol led to relevant increase of serum levels of concomitant AED, those antiepileptic drugs were decreased as clinically indicated. In case of study withdrawal, CBD was tapered down over 2–4 weeks.

### Efficacy Assessments

Terminology of patient's seizure types was synchronized between physicians and patients/caregivers prior to enrollment. Seizure frequency was recorded for all patients based on a prospective paper diary during a baseline of 4 weeks and during treatment. Parents were asked to document all visible seizures, but series of spasms were documented as one seizure. Motor seizures were defined as either focal or generalized seizures with a clear motor component of ≥3 s. Seizure diaries were reviewed by the study team at each clinic visit. A calculation of mean monthly seizure frequency was performed for baseline period and at each visit. In addition, the impact of CBD on seizures was measured by objective data from video-EEG at baseline and after 3 months of therapy, in all patients able to participate in monitoring. Video EEG was performed according to international guidelines over 24–72 h (14). During video-EEG monitoring, seizures were counted individually even if occurring in series. The number of seizures per 24 h was calculated accordingly.

The aim of the study was to establish safety and tolerability of synthetic cannabidiol. The primary endpoint was frequency of motor seizures at 3 months. For the main secondary efficacy analysis we assessed the monthly frequency of motor seizures at 6 and 12 months, as well as monthly frequency of all other seizure types including countable focal non-motor seizures but excluding myoclonic seizures and absences due to difficulties in counting them reliably. Those patients that did not characteristically exhibit countable seizures were not included in the primary endpoint analysis. We also examined the response rates for motor seizures and for all countable seizures, defined as patients whose reduction in mean monthly seizure frequency was >50%.

### Tolerability Assessments

Adverse events were documented by patients/caregiver, including epilepsy-related hospital admissions and emergency department visits and assessed at each visit and phone contact. Laboratory studies for hematology, electrolytes, liver and kidney function, and concentrations of antiepileptic drugs were conducted at baseline, and at every study visit.

### Statistical Analysis

The study sample size was not predetermined but based on patient enrollment. Statistical software GraphPad Prism, Inc. version 8.1.1 (California, USA, www.graphpad.com) as well as IBM SPSS Statistics version 23 were used for statistical analyses. As most of the seizure parameters were not normally distributed, non-parametric statistical procedures were applied. For the analysis across all assessment points, Friedman tests were conducted. Single comparisons across two assessment points were calculated using Wilcoxon tests. Comparisons of the dosages and responder rates or absolute change in seizure frequency from baseline to end point between adults and children were conducted with a Mann-Whitney *U*-test. The threshold of statistical significance was *p* < 0.05. Patients who exited the study were not included in any calculations for the upcoming visit. Main analyses were repeated using the last-observation-carried-forward (LOCF) principle. Furthermore, as some changes in medication occurred during the study period, separate analyses were conducted excluding patients with any change in concomitant anticonvulsive medication. Correlation of seizure reductions in VEEG and in patients' diary was assessed using a Spearman correlation.

## Results

### Study Population

**Figure 1:**
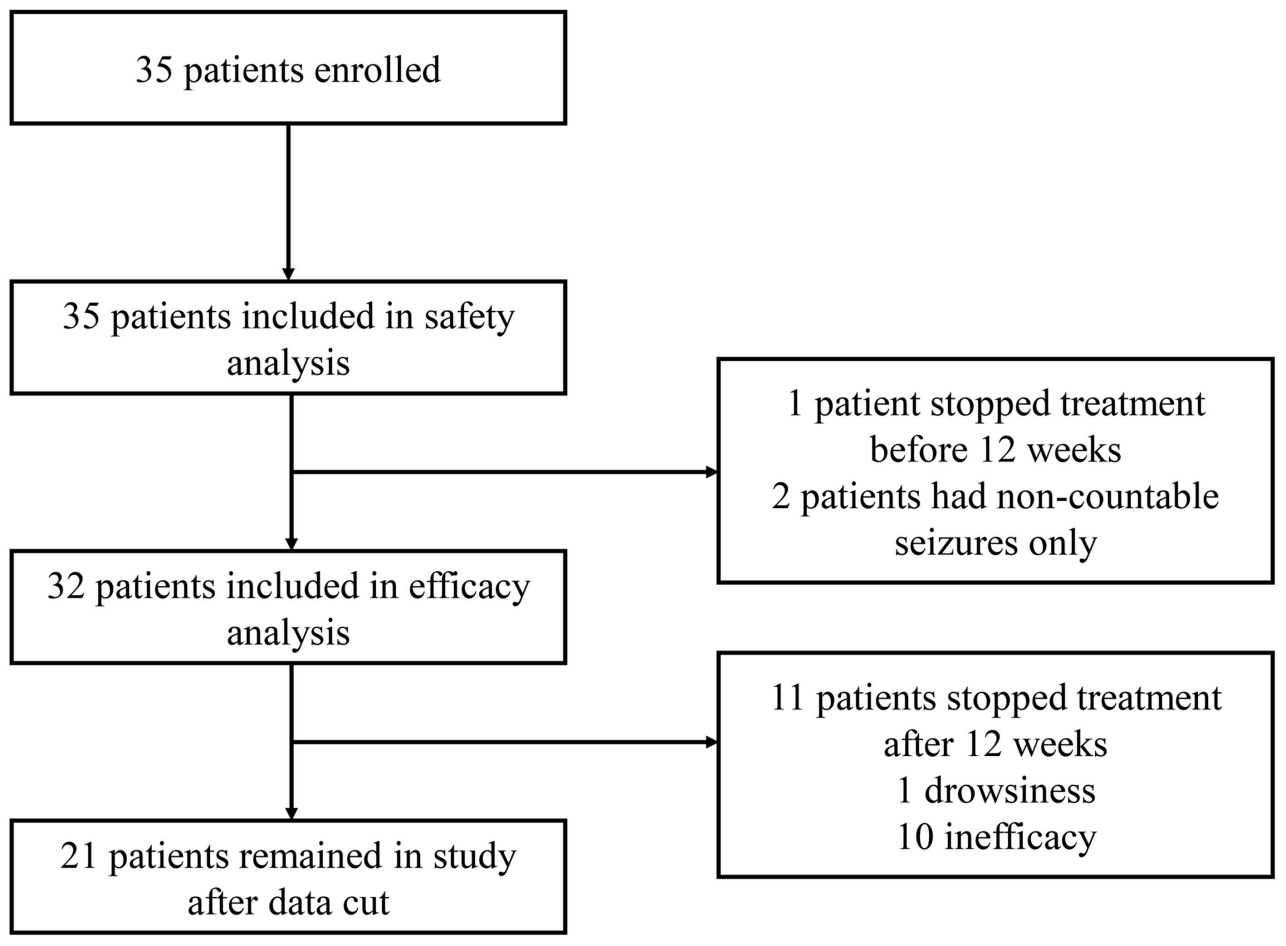
Study participants flow charts.

Between November 2017 and May 2019, 35 patients were enrolled in the study. All 19 children and all 16 adults were included in the safety analysis. In the efficacy analysis, 32 patients were included; two patients with uncountable seizures only; and one patient who withdrew due to adverse events after 4 weeks were excluded (Figure 1). Patient characteristics are shown in Table 1. Over 80% (*n* = 29) of the participants were taking a combination of at least two AED. The most commonly used medications were valproate (42.9%), lamotrigine (25.7%), and levetiracetam (22.9%). Clobazam was used by 4 patients (11.4%) in our study. By the time of data cut, 21 (60%) patients had ongoing treatment; the median CBD treatment duration was 321 days (range 36–824).

**Table 1:** Demographic and baseline characteristics.

	**Total (*n* = 35)**	**Children (*n* = 19)**	**Adults (*n* = 16)**
Male *n* (%)	19 (54.3)	10 (52.6)	9 (56.3)
Age in years mean (SD)	19.7 (14.6)	9.1 (4.7)	32.2 (12.2)
Age at epilepsy beginning median (range)	2 (0.08–52)	1 (0.08–11)	7 (0.33–52)
**Epilepsy syndrome** ***n*** **(%)**			
Focal/multifocal	15 (42.9)	5 (26.3)	10 (62.5)
Epileptic encephalopathy^*^	6 (17.1)	5 (26.3)	1 (6.25)
Lennox-Gastaut syndrome	6 (17.1)	4 (21.1)	2 (12.5)
Dravet syndrome	5 (14.3)	2 (10.5)	3 (18.75)
Doose syndrome	2 (5.7)	2 (10.5)	0
Generalized epilepsy	1 (2.9)	1 (5.3)	0
**Etiology** ***n*** **(%)**			
Genetic	18 (51.4)	11 (57.9)	7 (43.8)
Structural	15 (42.9)	8 (42.1)	7 (43.8)
Unknown	2 (5.7)	0	2 (12.4)
**Therapy**			
Concomitant AED median (range)	2 (1–4)	2 (1–3)	2 (1–4)
Previous AED median (range)	7 (2–23)	5 (2–12)	10 (2–23)
Previous ketogenic diet *n* (%)	11 (31.4)	9 (47.4)	2 (12.5)
Previous steroid treatment *n* (%)	10 (28.6)	10 (52.6)	0
Previous epilepsy surgery *n* (%)	2 (5.7)	1 (5.3)	1 (6.25)

* *Not otherwise specified. AED, anticonvulsive drug(s)*.

### Dosing

**Figure 2:**
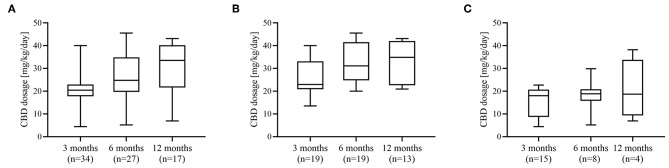
Dosage of cannabidiol. Dosage of CBD (mg/kg/day) administered at 3, 6, and 12 months of treatment in **(A)** whole cohort, **(B)** pediatric cohort, and **(C)** adult cohort.

By the 3 months follow up visit, titration to the target dose of 18 mg/kg/day CBD was achieved in 28 patients (80%). The remaining seven participants did not reach the target dose due to diarrhea (*n* = 4), excessive somnolence (*n* = 2), and elevated liver enzymes (*n* = 1). Titration to a maximum dose above 25 mg/kg per day at any time during the study was done in 12 children (63.2%) and one adult (6.3%). Six patients (17.1%) reduced their dose of CBD at any time during follow-up. The mean actual doses attained are shown in Figure 2. The median dose in children was significantly higher compared to adults at 3 months (*p* = 0.006) and at 6 months (*p* < 0.001).

### Efficacy

**Figure 3:**
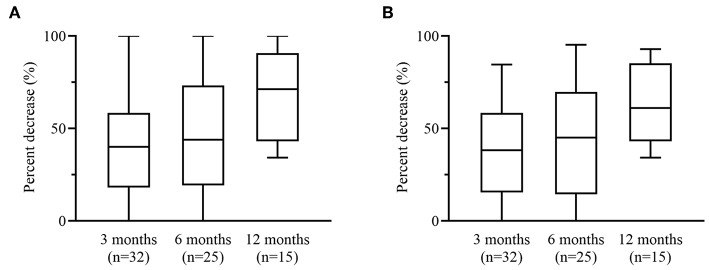
Percentage change of motor and all countable seizures. Median percentage change at 3, 6, and 12 months in **(A)** motor seizures and **(B)** all countable seizures. Boxplots show median value with 25th and 75th percentile. Whiskers denote minimum and maximum.

Median motor seizure frequency decreased from 21.8 (IQR 7.5–52.5) seizures per month at baseline to 8.5 (IQR 3.7–28.3, *p* < 0.001) at 3 months. Total seizure frequency decreased from a median of 22.3 (IQR 7.7–53.0) seizures per month at baseline to 11.85 (IQR 4.3–30.3, *p* < 0.001) at 3 months. Seizure reductions of motor and all countable seizures were statistically significant across all assessment points (both *p* < 0.001) and across individual assessment points (Table 2). Adjusted median reduction of motor seizures was 40.0% (IQR 18.2–58.5) and of all seizures 38.4% (IQR 18.6–58.9) at 3 months (Figure 3). One patient was free of all motor seizures during the 12 months treatment period. No patient reported an increase in countable seizure frequency, but one patient reported a subjective increase of absences.

**Table 2:** Frequency of motor seizures and of all countable seizures at baseline and at 3, 6, and 12 months of cannabidiol treatment in whole cohort, pediatric, and adult cohort.

**Seizure types and cohort**	**Visit**	***N***	**Median**	**Range**	***p***
**Motor seizures**					
All patients	Baseline	32	21.8	1.7–330	
	3 months	32	8.5	0–225	<0.001
	6 months	25	7.0	0–124.7	<0.001
	12 months	15	8.5	0–89.5	=0.008
Adults	Baseline	14	10.2	1.7–53.0	
	3 months	14	7.0	0.8–33.0	<0.001
	6 months	7	3.4	0.3–14.0	=0.06
	12 months	3	3.0	0–12.0	=0.05
Children	Baseline	18	42.0	1.7–330	
	3 months	18	13.0	0–225	<0.001
	6 months	18	11.7	0–224.7	<0.001
	12 months	12	7.5	0–89.0	=0.005
**All countable seizures**					
All patients	Baseline	32	21.3	1.7–330	
	3 months	32	11.9	1–225	<0.001
	6 months	25	11.7	0.7–124.7	<0.001
	12 months	15	7.5	0.5–89.0	=0.008
Adults	Baseline	14	12.2	2.7–53.0	
	3 months	14	7.2	1.0–33.0	=0.016
	6 months	7	15.0	2.0–28.7	=0.31
	12 months	3	3.0	0.7–18	=0.25
Children	Baseline	18	42.0	1.7–330	
	3 months	18	14.7	1.0–225.0	<0.001
	6 months	18	11.7	0.7–124.7	=0.005
	12 months	12	10	0.5–89.0	=0.001

*Significance level of seizure frequency at each visit compared to baseline*.

Results of the LOCF analysis showed that the observed reductions in seizure frequency were not affected by dropouts with a median reduction of monthly motor-seizure frequency compared to baseline of 40% (IQR 18.2–58.5, *p* < 0.001) after 3 months and 47.8% (IQR 16.4–76.8, *p* < 0.001) after 6 months. Also, the result of primary-endpoint result did not change when patients with any change in baseline AED therapy (*n* = 9) were excluded from the analysis (*p* < 0.001). Patients younger than 18 years at treatment onset had a significantly higher reduction in motor seizures than adults at 3 months (53.9 vs. 34.5%, *p* = 0.01). The percent reduction in motor seizures after 3 months was similar between those patients with Lennox-Gastaut or Dravet syndrome (*n* = 10) and those patients with other epilepsies (*n* = 22) (median 36.7 vs. 42.3%, *p* = 0.64). The ≥50% responder rate for motor seizures after 3 months was 43.0% and after 6 months 56.1%. Treatment response rates were generally similar in the LOCF analysis, with ≥50% responder rate for motor seizures of 43.8 after 3 months and 60% after 6 months. In those patients receiving video-EEG monitoring, seizure reduction at 3 months compared to baseline was statistically significant, both for frequency calculated from seizure diary (*p* = 0.001) and as recorded during monitoring (*p* = 0.01) (Table 3). There was, however, no clear correlation between seizure frequency measured by VEEG and by seizure diary at baseline (*R* = 0.407, *p* = 0.15) and at 3 months (*R* = 0.494, *p* = 0.075). The long-term retention rate after 6 and 12 months was 78.1 and 73.1% respectively.

**Table 3:** Percent reduction in seizures recorded during 48 h of video-EEG monitoring at baseline and after 3 months of CBD treatment compared to percent reduction if seizures as calculated from seizure diary in relation to patient's habitual seizures.

**Subject**	**Percent reduction of all seizure types**		**Patient's seizure types**
	**Recorded in VEEG**	**Calculated from diary**	
01	0	75.7	DS
02	40.5	57.1	ES, TS
03	66.7	NA	AS
04	38.6	54.9	ES
05	+78.0^*^	64.0	BTCS
06	15.3	7.7	BTCS, MS, AS
07	100	16.4	BTCS
08	100	72.5	Focal unaware non-motor
09	0	84.5	DS
10	85.6	19.9	ES, nocturnal TS
11	53.8	41.6	ES, focal unaware non-motor
12	0	38.4	Focal unaware non-motor
13	100	65.5	ES, nocturnal MS, BTCS
14	46.3	0	ES, TS
15	82.8	98.6	TS, focal non-motor, HKS

*AS, absences; BTCS, bilateral tonic clonic seizures; DS, drop seizures; ES, epileptic spasms; HKS, hyperkinetic seizures; MS, myoclonic seizures; NA, not applicable; TS, tonic seizures; VEEG, video-EEG monitoring*.

* *Increase in seizure frequency*.

### Adverse Events and Withdrawals

Twenty-five patients (71.4%) reported at least one adverse event (Table 4). Most adverse events were mild and transient. Possibly treatment related serious adverse events were reported in 2 (5.7%) patients and included one adult patient with drowsiness and one adult with extrapyramidal symptoms both requiring hospitalization. The latter occurred shortly after onset of omeprazole therapy and declined after omeprazole was stopped. Somnolence was reported in 14 patients (40%), of those only three patients received concomitant clobazam. Nine participants recovered without intervention; the cannabidiol dose was reduced, or titration interrupted in four patients, and in one patient clobazam dose was reduced. A significant weight loss of more than 5% from baseline was seen in four participants ranging from 7.5 to 20% of body weight. A significant weight gain of more than 5% from baseline was also observed in eight patients, ranging from 5 to 26.3% of body weight. There were no clinically significant changes in white or red blood cell counts, thrombocytes counts or renal function. In five patients occurred elevated ALT, AST, or GGT levels >3 times the upper limit of normal. Of those, three patients were taking valproate. Increased ALT/AST/GGT levels had resolved in four patients spontaneously and in one patient following treatment discontinuation. Altogether, two patients (5.7%) left the study due to adverse events after 4 and 12 weeks, respectively. Cumulatively, 10 (28.6%) patients withdrew due to lack of efficacy, of those, 6 between 3 and 6 months and 4 between 6 and 12 months.

**Table 4:** Adverse events observed during whole observation period.

**Adverse events**	**Total *n* (%)**	**Children *n* (%)**	**Adults *n* (%)**
At least one AE	25 (71.4)	16 (84.2)	9 (56.3)
Somnolence	14 (40)	11(57.9)	3 (18.8)
Diarrhea	12 (34.3)	7 (38.8)	5 (31.3)
Weight gain >5%	8 (22.9)	8 (42.1)	0 (0)
Loss of appetite	7 (20)	5 (26.3)	2 (12.5)
Irritability	7 (20)	6 (31.6)	1 (6.3)
Increased appetite	5 (14.3)	4 (21.1)	1 (6.3)
Weight loss >5%	4 (11.4)	2 (10.5)	2 (12.5)
Others^*^	4 (11.4)	4 (21.1)	0 (0)

* *drowsiness (n = 3), extrapyramidal symptoms (n = 1)*.

*AE, adverse event*.

### Co-medication and Interaction

In 9 patients, AED levels over the upper therapeutic range or increase of levels >10% were detected. In three of four patients on clobazam, the active metabolite desmethylclobazam (D-CLB) increased by 20–468%. The only patient on clobazam with no change in D-CLB level was also on primidone. Of seven patients on brivaracetam (BRV), four patients showed an increase of brivaracetam plasma levels by 107–280%. In the remaining three patients on BRV, the levels were not available. In one patient on eslicarbazepine, the level increased by 23%, whereas in the only other patient on eslicarbazepine, no change was seen.

During the whole observation period, three patients stopped one of the concomitant AED, three patients started a new AED, and 30 patients remained on the initial comedication. Of those 30 patients, 26 also remained on the initial dose, whereas in two patients, dose of concomitant AED was reduced due to an increase in plasma levels and in two patients, dosage was increased as an effort to optimize treatment.

## Discussion

In our prospective open-label study, add-on treatment with synthetic cannabidiol led to a clinically meaningful reduction in seizure frequency in many patients and had an adequate safety profile in this patient population of children and adults with highly treatment-resistant epilepsy. Eighty percent of our patients were treated with two or more anticonvulsive drugs at baseline but still had a high seizure frequency with a median of almost 22 motor seizures per month.

Median modal dose after 3 months of treatment was similar for adults and children about 20 mg/kg/days and comparable with dosages in studies using plant derived cannabidiol (15). Whereas, adults remained on a stable median dose over the whole treatment period, dose was increased in most children between 3 and 6 months, resulting in a broader dose range. We believe this reflects a higher metabolism and a higher tolerance in children, rather than a secondary loss of efficacy. Other studies with longer observation periods also reported maintenance of long term efficacy (16, 17).

The present study showed a statistically significant reduction in motor seizures. Primary endpoint was frequency of monthly motor seizures at 3 months, which decreased significantly compared to baseline by an adjusted percentage reduction of 40%. At 6 months the median percentage change in the monthly frequency of motor seizures was 49.3%. The increase in treatment response is not explained by drop-outs, but rather reflects a higher fraction of children in the 6 months cohort and might also be related to a better dose-finding after 6 months compared to 3 months. As this is an ongoing study, numbers of patients receiving treatment for more than 12 months are too small to assess the long-term efficacy. Nevertheless, retention rates at 6 and 12 months are 78.1 and 73.1%, respectively, and comparable with other open label studies using plant-derived CBD (18). The role of clobazam-cannabidiol interaction in seizure reduction has been discussed before and is not fully understood (1). In our study, the rate of clobazam comedication (11%) was much lower but responder rates and median percent reduction of seizures similar compared to other open label studies (6). Seizure frequency reduction was significantly higher in children than in adults. This difference could be based on a shorter disease duration, but numbers were too small to assess confounding factors in treatment response in our study. In larger open label studies, efficacy for children and adults is not reported separately (6). The better treatment response might also be explained by the much higher monthly seizure frequency at baseline in children (median 42) compared to adults (median 12.2). Etiology might be another important point in treatment response since more than 60% of the children had epileptic encephalopathy whereas more than 60% of adults had focal/multifocal epilepsy. Besides the randomized controlled trials, only few studies assessed CBD efficacy in specific epilepsy etiologies or syndromes, but data are not sufficient to support the perception of a better treatment response in children with epileptic encephalopathy (19). In our cohort as well, we could not see statistically significant differences in a direct comparison of patients with Lennox-Gastaut or Dravet syndrome vs. patients with other epilepsies.

Synthetic cannabidiol was generally well-tolerated. As in previous open label studies using plant-derived CBD, adverse events were reported frequently but remained mainly mild or moderate; only two patients discontinued treatment due to adverse events. The most common side effects in our study were similar to those reported previously: somnolence (40%) and diarrhea (34.3%) (20). Another frequently described side effect in long term open label studies is loss of appetite, resulting in weight loss in some patients (16). Interestingly, whereas loss of appetite was also reported in our study, overall more patients showed a significant weight gain (22.9%) than a significant weight loss (11.4%). Especially in children with epileptic encephalopathies and a high seizure burden, the general condition improved considerably, permitting an improved nutrition and likely explaining a weight gain in 40% of those children. As reported before, significant increase of liver enzymes was more frequent in patients taking valproate, but also observed in patients without any comedication (20). In our cohort, patients and parents did not report any significant changes in behavior or concentration. However, long-term effects on cognition is one major concern with cannabinoid treatment (21). Longer observational periods and studies with a more detailed neurocognitive test protocol are needed.

Overall, 25% of patients in our study showed a relevant increase of their concomitant AED's plasma levels, in particular desmethylclobazam. Increased sedation in those patients is in line with previous reports, but occurred in patients without clobazam also (22). The only patient with no increase in D-CLB level was also on primidone, a potent inducer of Cytochrome P450 (CYP) enzymes, counteracting the CYP-enzyme inhibiting effect of CBD. However, we also found interaction with other AED, such as BRV that is described in detail elsewhere, and potential interaction with other comedications as omeprazole (23). Cannabidiol is known for its high interaction potential but further pharmacokinetic studies are needed to understand clinically relevant interactions with AED and other drugs in mono-and polytherapy (24).

There are several limitations in this study. As an open-label study of a drug with high public interest, the placebo-effect may be augmented compared to other anticonvulsive drugs. However, the overall median reduction of motor-seizures in our study was comparable to other open label studies as well as randomized controlled trials using plant-derived CBD (15). Although doses and number of AED remained stable in the majority of patients, another limitation is that concomitant AED were not strictly controlled. In any case, the calculations that include only patients with stable AED regimen still revealed statistically significant reductions in seizures, making a relevant contribution of AED changes to the outcome in this study unlikely. Also, our cohort was very heterogeneous, limiting the assessment of efficacy in this population.

Even though it is generally accepted to calculate primary outcome variables from patients' seizure diaries, there is distinct evidence that patient-reported seizure-counts lack validity (25). In our study, seizure reduction as observed by video-EEG (video-efficacy) and seizure reduction as calculated from seizure diaries (diary-efficacy) showed discrepancy in 5 of 15 patients. As expected, mainly nocturnal seizures and seizures without prominent motor signs were present in those patients. Reduction of video-EEG-recorded seizures was statistically significant after 3 months compared to baseline (*p* = 0.01), showing efficacy of cannabidiol in our cohort when established in an objective manner. Reduction of seizures of those patients as calculated from seizure diary was still highly significant (*p* = 0.001). The difference between video-efficacy and diary-efficacy could be based on a placebo effect in CBD treatment. However, the comparison is limited by the different time frames of both methods and there is no clear statistical correlation between these two measures. An additional benefit of video-EEG monitoring was the possibility to reveal a significant seizure reduction in one patient who only exhibited non-countable seizure and was therefore not included in the primary outcome measurement. Counting seizure frequency in an objective manner during video-EEG is time- and cost-consuming and not possible in all patients but might be a sensitive approach in patients with subtle and nightly seizures at least.

The exact mechanism of action of CBD has been only partially elucidated and involves a series of different mechanisms but has been shown to be mainly independent of the endocannabinoid system (26). For other indications than epilepsy, combinations of cannabinoids have been used and the entourage effect, i.e., the positive synergistic effect of different cannabis compounds has been discussed. Despite some metanalytic evidence of potential clinical benefits of CBD extracts over purified CBD, informative value is extremely limited by quality of the included studies (27). In almost all prospective open label studies with larger cohorts purified plant derived CBD has been used. Since pharmaceutical-grade synthetically derived cannabidiol is chemically identical to the cannabidiol found naturally in the cannabis plant, we did not expect any relevant differences in efficacy and tolerance (10). Nevertheless, this study is the first to report efficacy and tolerance of synthetic cannabidiol in epilepsy treatment to be comparable to plant derived CBD. The production of plant derived CBD is economically and ecologically challenging, and it is unclear if will be possible to meet increasing global demand for the long term. Therefore, synthetic CBD might be a worthwhile alternative.

## Conclusion

In summary, the results of this study provide class III evidence of efficacy and safety of synthetic cannabidiol in children and adults with pharmacoresistant epilepsy. Additional studies investigating efficacy and tolerance of synthetic CBD in larger cohorts are needed.

## Data Availability Statement

The raw data supporting the conclusions of this article will be made available by the authors, without undue reservation, to any qualified researcher.

## Ethics Statement

The studies involving human participants were reviewed and approved by Ethik-Kommission der Albert-Ludwigs-Universität Freiburg, Germany. Written informed consent to participate in this study was provided by the participants' legal guardian/next of kin.

## Author Contributions

KK designed and conceptualized study, analyzed the data, and drafted the manuscript for intellectual content. DG played major role in the acquisition of data. BM interpreted the data, revised the manuscript for intellectual content, and performed statistical analysis. MH, AS-B, and JJ interpreted the data and revised the manuscript for intellectual content.

## Conflict of Interest

AS-B received honoraria for lectures and advice from BIAL, EISAI, Precisis, and UCB. The remaining authors declare that the research was conducted in the absence of any commercial or financial relationships that could be construed as a potential conflict of interest.
